# Nondestructive and Rapid Screening of Aflatoxin-Contaminated Single Peanut Kernels Using Field-Portable Spectroscopy Instruments (FT-IR and Raman)

**DOI:** 10.3390/foods13010157

**Published:** 2024-01-02

**Authors:** Siyu Yao, Gonzalo Miyagusuku-Cruzado, Megan West, Victor Nwosu, Eric Dowd, Jake Fountain, M. Monica Giusti, Luis E. Rodriguez-Saona

**Affiliations:** 1 Department of Nutrition and Food Hygiene, School of Public Health, Southeast University, Nanjing 210009, China; 2 Department of Food Science and Technology, The Ohio State University, Parker Food Science and Technology Building, 2015 Fyffe Road, Columbus, OH 43210, USAgiusti.6@osu.edu (M.M.G.); rodriguez-saona.1@osu.edu (L.E.R.-S.); 3 Mars Wrigley, Inc., 1132 W. Blackhawk Street, Chicago, IL 60642, USAeric.dowd@effem.com (E.D.); 4 Department of Plant Pathology, University of Georgia, 216 Redding Building, 1109 Experiment St., Griffin, GA 30223, USA

**Keywords:** aflatoxin, FT-IR, Raman, field-portable instruments, chemometrics, food safety control

## Abstract

A nondestructive and rapid classification approach was developed for identifying aflatoxin-contaminated single peanut kernels using field-portable vibrational spectroscopy instruments (FT-IR and Raman). Single peanut kernels were either spiked with an aflatoxin solution (30 ppb–400 ppb) or hexane (control), and their spectra were collected via Raman and FT-IR. An uHPLC-MS/MS approach was used to verify the spiking accuracy via determining actual aflatoxin content on the surface of randomly selected peanut samples. Supervised classification using soft independent modeling of class analogies (SIMCA) showed better discrimination between aflatoxin-contaminated (30 ppb–400 ppb) and control peanuts with FT-IR compared with Raman, predicting the external validation samples with 100% accuracy. The accuracy, sensitivity, and specificity of SIMCA models generated with the portable FT-IR device outperformed the methods in other destructive studies reported in the literature, using a variety of vibrational spectroscopy benchtop systems. The discriminating power analysis showed that the bands corresponded to the C=C stretching vibrations of the ring structures of aflatoxins were most significant in explaining the variance in the model, which were also reported for *Aspergillus*-infected brown rice samples. Field-deployable vibrational spectroscopy devices can enable in *situ* identification of aflatoxin-contaminated peanuts to assure regulatory compliance as well as cost savings in the production of peanut products.

## 1. Introduction

Peanut (*Arachis hypogaea* L.) is an important cultivated crop globally, grown in over 100 countries, with a production of more than 40 million tons every year [1,2]. As a rich source of nutrients, peanut provides protein, lipids, carbohydrates, and essential amino acids to the human body [3]. However, toxigenic fungi are ubiquitous in nature and can infect vulnerable agricultural commodities such as peanuts [4]. The secondary metabolites of those fungi, named mycotoxins, can induce a wide range of toxic activities, ranging from acute intoxication to long-term effects such as immune deficiency and cancer [5,6]. Mycotoxin contamination can occur in the field and during any point in the postharvest stages (i.e., food production and storage) under favorable conditions [7,8]. Adequate agricultural practices, good storage conditions, and timely plant disease management limit mycotoxin contents in the food supply chain but cannot eliminate mycotoxin contamination completely [7].

Among the thousands of mycotoxins in existence, the most well known are aflatoxins, which are primarily produced by *A*. *flavus* and *A. parasiticus* strains. Aflatoxin B_1_, B_2_, G_1_, and G_2_ are the most toxic and carcinogenic *Aspergillus* metabolites found in peanuts [9]. *A. flavus* can produce aflatoxin B_1_ and B_2_, while *A. parasiticus* is able to produce all four types [1]. Considering the hazards posed to health by aflatoxins and ensuring the safety of food and agricultural products, many countries have established mandatory regulations on aflatoxin content. For example, the European Commission (EU) has set aflatoxin B_1_ contamination limits as less than 5 ppb in food products (Regulation (EU) No. 165/2010]. In the United States, the Food and Drug Administration (FDA) set the maximum level of aflatoxin level at 20 ppb for food and 300 ppb for feed [10].

Numerous methods for assessing fungal-contaminated crops have been developed to confirm that aflatoxin levels remain below regulatory limits, such as thin-layer chromatography [11], high-pressure liquid chromatography [12], immunoaffinity columns [13], and enzyme-linked immunosorbent assays [14,15]. However, the laborious, slow, complex, and expensive sample preparation required for these analytical methods limits the nondestructive and real-time assessments of the large number of peanuts consumed every day [16]. Additionally, aflatoxin contamination is often not uniform in a batch, with a few contaminated peanut kernels accumulating high levels of toxins, posing a safety risk and resulting in the need for single kernel measurement [17]. Therefore, a real-time and sensitive approach to continually screen single peanuts and detect mycotoxins could provide assurances of regulatory compliance as well as cost savings in the complicated food supply network [8].

Vibrational spectroscopic techniques such as Raman spectroscopy and infrared spectroscopy (IR) are powerful fingerprinting techniques used in metabolomics, enabling the nondestructive, rapid, and high-throughput assessment of a broad variety of metabolites [18]. These techniques are based on the transitions between the quantized vibrational energy states of molecules due to the transition between the radiation from a light source and the sample material [19]. IR and Raman are complementary to each other, since, for a molecule to be IR active, the dipole moment of the molecule has to change for antisymmetrical vibrations. For Raman scattering to be active, the polarizability needs to be changed for symmetric vibration [20]. Driven by advancements in micro electromechanical systems (MEMS) and optoelectronics, portable/handheld Raman and IR spectrometers have shown spectral resolution and precision equivalent to those of benchtop instruments, which have the potential to be applied in the field without requiring complicated laboratory environment [21].

Few applications related to utilizing vibrational techniques for aflatoxin screening in peanuts have been reported. Benchtop FT-IR was applied to discriminate aflatoxin from an extracted peanut oil fraction [22,23], and solvent-extracted ground peanut cake [24] and surface-enhanced Raman spectroscopy (SERS) techniques have been reported for the quantitative detection of aflatoxin in peanuts [25,26]. However, these SERS studies were constrained by the required pretreatment extractions and poor reproducibility in field applications [27]. With respect to utilizing portable vibrational techniques (FT-IR and Raman), no scientific papers have been found in the literature dealing with the ability to perform nondestructive and rapid detection of aflatoxin on single peanut kernels.

Herein, our objective in this research was to evaluate the performance of portable vibrational spectroscopy (FT-IR and Raman) combined with pattern recognition analysis for the nondestructive and rapid detection of single aflatoxin-contaminated peanut kernels, potentially allowing for real-time and in situ screening of peanuts to support breeding programs, postharvest research, and regulatory compliance in the future.

## 2. Materials and Methods

### 2.1. Sample Preparation

The aflatoxin mix standard (aflatoxin B_1_: 1 μg/mL, aflatoxin B_2_: 3 μg/mL, aflatoxin G_1_: 1 μg/mL, and aflatoxin G_2_: 3 μg/mL, in benzene:acetonitrile (98:2), a certified reference material) was purchased from Sigma-Aldrich (St. Louis, MO, USA). Aflatoxin-free shelled peanuts (variety: Tifrunner) were obtained from the USDA (Crop Genetics and Breeding Research Unit, Tifton, GA, USA). Hexane, HPLC-grade, was collected from Thermo Fisher Scientific (Waltham, MA, USA). For the Raman study, a total of 90 peanuts with skins (average weight of 0.7 g) were chemically spiked with aflatoxins, applying 10 μL of a hexane solution containing different concentrations of the aflatoxin mix on one side of each peanut, achieving target total aflatoxin concentrations of 30, 40, 50, 100, 200, and 400 parts per billion (ppb). Target concentrations of the aflatoxin mix were prepared via evaporating different volumes of the aflatoxin mix standard (1 µg/mL B_1_, 3 µg/mL B_2_, 1 µg/mL G_1_, 3 µg/mL G_2_) using a sample concentrator (BTLab Systems, St. Louis, MO, USA), followed by redissolving the mix with a constant volume (10 μL) of hexane. The opposite side of each peanut served as control, to which we applied with 10 μL of hexane. In the FT-IR study, since the attenuated total reflectance (ATR) involves applying pressure to create good contact between the peanut and the ATR crystal, this pressure resulted in minor oil being pressed out from kernels. The strong oil signal in the IR spectra could mask the fingerprinting information from aflatoxin and decrease the discrimination ability of the algorithms with the IR analysis. Thus, FT-IR measurement was only conducted using different peanuts for spiking and controls. We spiked 60 peanuts with aflatoxins (30–400 ppb) using the same approach described above, while another 60 peanuts were spiked with 10 μL of hexane to serve as controls. After spiking, all peanuts with skins were placed under a chemical hood for 10 min to allow the solvent to evaporate and enable aflatoxins to be well absorbed into the peanut before spectral acquisition. In addition, two peanuts without any chemical treatment were randomly selected to characterize the spectral information of peanut cotyledons, and their skins were removed before FT-IR and Raman spectral acquisition.

### 2.2. FT-IR and Raman Spectral Acquisition

The FT-IR spectral acquisition was conducted using a portable FT-IR spectrometer, 4500a (Agilent Technologies, Santa Clara, CA, USA), equipped with a 3-reflection diamond attenuated total reflectance (ATR), a thermoelectrically cooled deuterated triglycine sulfate (DTGS) detector, and a Michaelson interferometer (disperse light). The spectrometer had a 2 mm diameter sampling surface, with a 200 μm active area providing a 6 μm effective penetration depth for IR energy at 1700 cm^−1^. The peanut FT-IR spectra were collected via applying a uniform pressure on each peanut surface to cover the active sampling area, using a high-pressure clamp accessory. Spectral data were collected from 4000 to 700 cm^−1^ with a resolution of 4 cm^−1^. A total of 64 scans (~1 min) were co-added for each spectrum collection to obtain an excellent signal-to-noise ratio, and a background was collected before each spectrum collection to eliminate the environmental variations.

Raman spectral data were collected from 200 to 2500 cm^−1^ using a Progeny^TM^ handheld Raman spectrometer (Rigaku Analytical Devices, Wilmington, MA, USA) with a 1064 nm excitation wavelength and an InGaAs array detector, and the system was operated with a spectral resolution of 4 cm^−1^. A tablet adapter was used to hold the single kernel for the measurement. The laser power was set to 490 mw, and the exposure time was set to 2.5 s, with 20 averages to maximize the signal-to-noise ratio and avoid burning of the peanut kernels. All spectral data from FT-IR and Raman were collected once on each single kernel to avoid potential cross-contamination during the operation.

### 2.3. Aflatoxin Spiking Verification Analysis (Ultra-High-Performance Liquid Chromatography–Tandem Mass Spectrometry (uHPLC-MS/MS) Analysis) of Peanuts

To ensure the desired content of aflatoxin was spiked onto the peanuts, 16 peanuts spiked with 50, 100, 200, and 400 ppb were randomly selected after spectral acquisition to verify the aflatoxin content. An uHPLC-MS/MS method with Quick Easy Cheap Effective Rugged and Safe (QuEChERS) pretreatment was used with some modifications [15,28,29,30]. A single peanut kernel was placed into a 50 mL centrifuge tube; 10 μL of aflatoxin M_1_ (10 μg/mL, Sigma-Aldrich, St. Louis, MO, USA) was added as internal standard (IS); and 20 mL of hexane was added and blended using a hand homogenizer (D100, Benchmark Scientific Inc, Sayreville, NJ, USA) in the tube. Then, 12.9 mL of acetonitrile and 2.1 mL of water with 0.1% formic acid (Sigma-Aldrich, St. Louis, MO, USA) were added to each tube, which was shaken vigorously for 20 min at 80 rpm. After that, a salt mixture from the QuEChERS kits (Restek, Bellefonte, PA, USA) containing 4 g of MgSO_4_, 1 g of TSCD, 1 g of NaCl, and 0.5 g of DHS was added to the tube, which was further shaken vigorously for another 20 min at 80 rpm, and then centrifuged for 40 min at 4000 rpm at 4 °C. The top hexane layer was removed to remove the fat from the sample, and 12 mL of the acetonitrile layer was transferred to a QuEChERS dSPE tube (1200 mg MgSO4, 400 mg PSA and 400 mg C18). dSPE tube was vortexed for 3 min and centrifuged for 15 min at 4000 rpm. The supernatant solution (7.2 mL) was completely dried via flushing with nitrogen gas, redissolved with 0.2 mL acetonitrile, filtered through a 0.22 µm nylon filter, and transferred into an autosampler vial with a target polypropylene conical insert (Thermo Fisher Scientific, Waltham, MA, USA) for LC-MS/MS analysis.

The total aflatoxin (B_1_, B_2_, G_1_, G_2_) levels on a single peanut surface were determined using a Nexera-i LC2040C 3D ultra HPLC coupled with a LCMS-8040 triple quadrupole mass spectrometer with electrospray ionization (UHPLC-ESI-MS/MS) (Shimadzu, Columbia, MD, USA). A reverse-phase Raptor ARC-18 column equipped with a guard column from Restek (Bellefonte, PA, USA) (2.7 µm particle size and 150 × 3.0 mm dimensions) was used for chromatographic separation. The injection volume and flow rate and were set at 5 μL and 0.3 mL/min, respectively, for each measurement. The separations were achieved using water with 2 mM ammonium formate and 0.1% formic acid as solvent A, and methanol with 2 mM ammonium formate and 0.1% formic acid as solvent B, with the following gradient: 35% B at 0.01 min, 35–65% B at 0.01–10 min, 65–35% B at 10–12 min, 35% B at 12–15.01 min. The mass spectrometer was operated in positive mode, and the time-managed multiple reaction monitoring (MRM) detection mode was utilized to identify the compounds using the mass-per-charge ratio of 313.10 > 241 and 313.10 > 213 for aflatoxin B_1_, 315.10 > 259.10 and 315.10 > 287 for aflatoxin B_2_, 329.10 > 243 and 329.10 > 200 for aflatoxin G_1_, 331.10 > 245 and 331.10 > 189 for aflatoxin G_2_, and 329 > 273 and 329 > 259 for aflatoxin M_1_ (IS). The quantification of aflatoxins was achieved using the calibration curves developed by plotting concentrations of the external standards and the area ratios of the external and internal standard. The aflatoxins levels in each sample were analyzed in duplicate via LC-MS/MS analysis.

### 2.4. Multivariate Data Analysis

Given the abundance of information within spectral data, multivariate data analysis is required to extract meaningful information [31]. Principal component analysis (PCA), an unsupervised technique, was performed to explore the natural clustering differentiating the control peanuts and the peanuts with aflatoxins. However, soft independent modeling of class analogy (SIMCA), a supervised technique, was relied to develop predictive classification algorithms that can be used to assign classes to unknown samples.

SIMCA uses the known knowledge about the classification information of samples, such as control peanuts (class 1) and peanuts with aflatoxins (class 2), to build up the training models in order to predict new unknown samples into known classes. Each class (category) in SIMCA is modeled independently using a PCA model, described by the different numbers of principal components. As a PCA model is generated for each class, SIMCA can provide information about the relevance of variables and outlier detection. When an unknown sample is projected into each PCA model, its degree of fit allows determining if this sample can be assigned to a unique class, fit to numerous classes, or just does not fit in any [31].

During the development of the FT-IR SIMCA aflatoxin detection model, 100 peanuts (50 peanuts with aflatoxins and 50 peanuts without aflatoxins) were randomly selected for developing a training model, and 20 peanuts (10 peanuts with aflatoxins and 10 peanuts without aflatoxins) served as an external validation set. Because of the limited capability of Raman spectra for discriminating peanuts spiked with aflatoxins below 100 ppb, the final Raman model was developed by including spiked peanuts with aflatoxin levels ≥ 100 ppb (100, 200 and 400 ppb). Thus, the number of peanuts in the training model included 105 Raman spectra (32 peanut spectra (≥100 ppb) containing aflatoxin and 72 Raman spectra (from the control side). The external validation set included 26 Raman peanut spectra (8 spectra collected from peanuts spiked with aflatoxin (≥100 ppb) and 18 Raman spectra collected from the control side) that were randomly selected and were not part of the training set. The training set was utilized to “train” the system to differentiate the unique aflatoxin fingerprinting profiles on the peanut surface from common FT-IR and Raman spectral features, which was accomplished by providing the known class assignments. The external validation set allows for evaluating the performance (i.e., accuracy, selectivity, and specificity) of SIMCA training models, generating an unbiased estimation of predictive performance on deploying the models in real applications. The performance of the SIMCA training model was evaluated in terms of interclass distance (quantitatively describing the dissimilarity and similarity; generally when ICD > 3, it is accepted as well differentiated), discriminating power (how well a variable discriminates between two classes), misclassifications (the percentage of samples correctly assigned into their original group), class projections, accuracy, selectivity, and specificity [32,33].

FT-IR and Raman spectral data were exported from the instruments as GRAMS (spc.) files and analyzed using chemometrics software, Pirouette^®^ (Version 4.5, Infometrix, Inc., Bothell, WA, USA). FT-IR spectra were preprocessed using the second derivative and smoothing (Savitzky–Golay polynomial fitting algorithm with a 25-point window) to remove the nonlinear background signal and mean centering to remove the intercept from the model and constant background noise prior to multivariate analysis [34]. Raman spectra were preprocessed via normalization (sample 2-norm) to take care of disparity in intensity levels and mean centering prior to multivariate analysis [35].

## 3. Results and Discussion

### 3.1. Pattern Recognition Model Development for Detecting Aflatoxin Contamination

**Figure 1:**
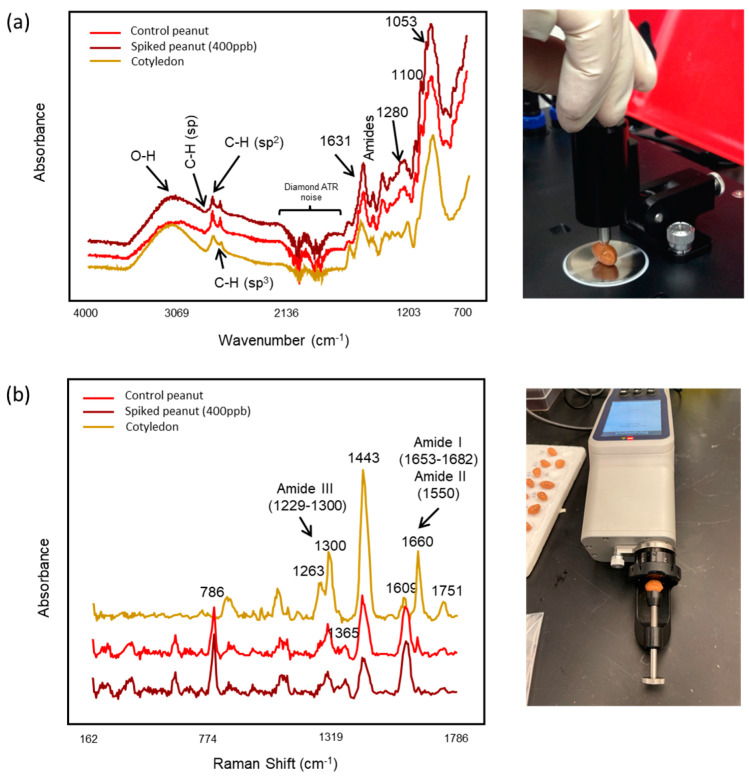
Representative raw spectra of a control peanut, a peanut with aflatoxins (400 ppb), and a pair of peanut cotyledons collected using (**a**) a portable FT-IR spectrometer and (**b**) a handheld Raman instrument (1064 nm).

The spectra obtained from a control peanut, a peanut with aflatoxins (400 ppb), and a pair of peanut cotyledons using triple-reflection ATR FT-IR are shown in Figure 1a. The midinfrared region showed distinct bands related to the components of the peanuts. The wide band centered at around 3200 cm^−1^ was associated with the water in the peanuts [36]. Absorbances at 3009–2800 cm^−1^ were related to =C-H *cis* stretching, -C-H symmetric, and asymmetric stretching vibrations of the lipid in peanuts [37,38]. The bands at 1640 cm^−1^ and 1542 cm^−1^ were associated with the amide I and II bands, which are typically bonds of C-N, N-H, C=C, and the combination of N-H and C-H (amide II) related to protein amino acids [39]. The strong absorption bands located at 1013 cm^−1^, 1080 cm^−1^, and 1052 cm^−1^ corresponded to the organic acids and carbohydrates [9]. Spectra collected from a control peanut and a peanut with aflatoxins (400 ppb) showed close similarity in their spectral characteristics. The spectral differences between cotyledons and peanuts with skin (control peanut and peanut with aflatoxins) were mainly located at 1053 cm^−1^, 1100 cm^−1^, 1280 cm^−1^, and 1631 cm^−1^. The bands centered at 1053 cm^−1^, 1100 cm^−1^, and 1280 cm^−1^ were unique for the spectra obtained from peanuts with skin, which were related to the asymmetric stretching of the C-O pyranose, C-O-C from β-1,4 glycosidic linkages, and CH bending in the cellulose structure [40]. The band located at 1631 cm^−1^ (O-H bending) was higher for peanut cotyledons because of the higher amounts of moisture content existing in cotyledons [41].

Raman spectra (cotyledons, control peanuts, and aflatoxin-spiked peanuts) exhibited unique features (Figure 1b). Several of the bands in the peanut spectra (cotyledons, control, and aflatoxin spiked) were similar. The band at 1751 cm^−1^ was associated with C=O stretch in aldehydes, carboxylic acids, and ketones; the band at 1660 cm^−1^ was related to C=C stretching vibrations and NH_3_^+^ and COO^−^ asymmetrical stretching vibrations related to unsaturated fatty acids and proteins [42]. Furthermore, the vibrational bands at 1653–1660 cm^−1^, 1550 cm^−1^, and 1229–1300 cm^−1^ corresponded to amide I (α helix and β sheet), amide II, and amide III, respectively [43,44]. Vibrational bands at 964 cm^−1^, 1286 cm^−1^, and 1443 cm^−1^ were associated with CH_2_/CH_3_ vibrations (aliphatic groups) [44]. However, some spectral dissimilarities, such as different intensities and bands, were observed between cotyledons and peanuts with skin. The bands centered at 1660 cm^−1^, 1443 cm^−1^, 1300 cm^−1^, and 1263 cm^−1^ had pronounced higher intensities for peanut cotyledons, which indicated higher contents of fatty acids and protein. The band located at 1609 cm^−1^ was higher for the peanuts with skin than cotyledons, which was attributed to the coupling effects of aromatic C=C stretch and NH_3_^+^ and COO^−^ asymmetrical stretching, indicating the presence of pigments on the peanut skin. The main unique bands in the spectra collected from peanuts with skin were centered at 1365 cm^−1^ and 786 cm^−1^, which corresponded to the polyphenols and organic acids on the skin [42,45].

**Figure 2:**
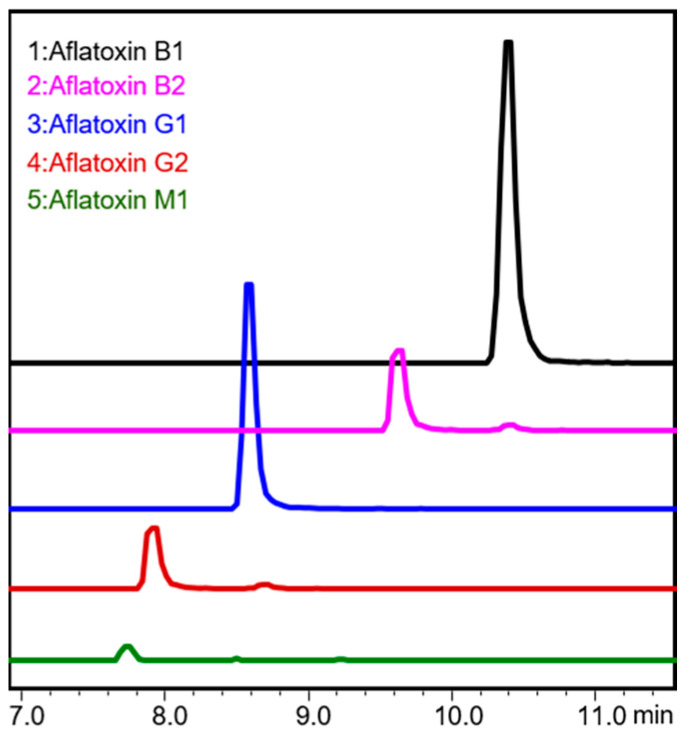
uHPLC-MS/MS representative spectra of aflatoxins.

After Raman and FT-IR spectral acquisition, 16 peanuts (50–400 ppb) were randomly selected and analyzed using uHPLC-MS/MS to ensure that the desired content of aflatoxin was spiked on the surface of the peanut kernels. Aflatoxins (B_1_, B_2_, G_1_, and G_2_) were identified and quantified based on their retention times and MS/MS spectra (Figure 2). The uHPLC-MS/MS results of the peanuts under each concentration generally matched their corresponding target spiked level, with minor standard deviations and standard errors, as shown in Table 1, which can facilitate the development of robust and accurate supervised predictive models.

Overall, both portable FT-IR and Raman instruments were able to capture some unique characteristics from the kernel skins, suggesting that these techniques have the potential to detect aflatoxins on a kernel’s surface. However, the FT-IR spectra provided more unique fingerprinting information/bands from the kernel skins than from the cotyledons compared with Raman according to the results of visual inspection. This is because FT-IR instruments equipped with an ATR accessory have a shallower penetration depth (~2 μm) than Raman systems (penetration depth at mm level, 1064 nm) [46,47]. In addition, it was difficult to identify a unique marker band associated with aflatoxin by visually inspecting the FT-IR and Raman spectra, mostly because of the overlapping bands from other predominant chemical components on the peanut surface and cotyledons. Therefore, the application of pattern recognition methods was critical for extracting spectral features to classify the peanuts contaminated with aflatoxins [9].

**Figure 3:**
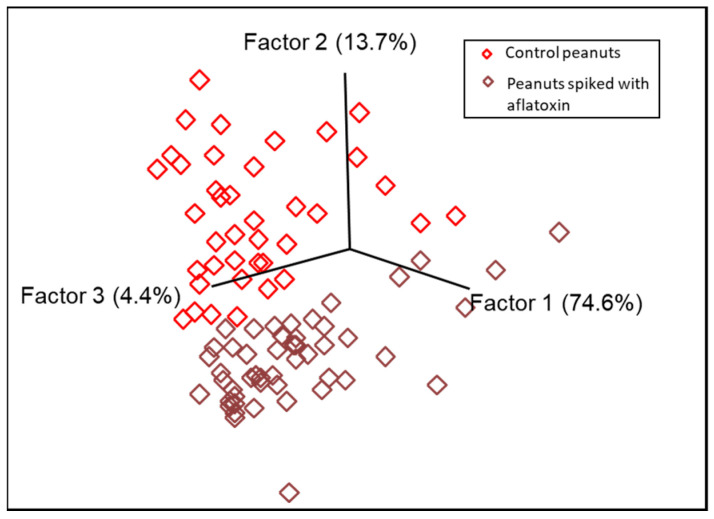
PCA score plot generated from the spectral data of peanut samples collected with a portable FT-IR spectrometer.

**Figure 4:**
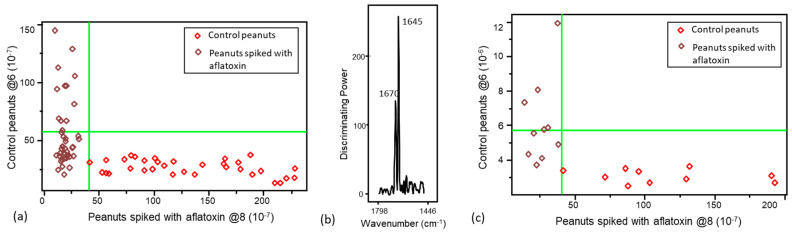
(**a**) Soft independent modeling of class analogy (SIMCA) Coomans plot and (**b**) SIMCA discriminating plot generated from the spectral data of peanut samples collected with a portable FT-IR spectrometer, and (**c**) the SIMCA Coomans plot for its corresponding external validation set.

PCA was utilized as an initial step before applying supervised pattern recognition methods. Results from PCA analysis demonstrated a natural clustering, differentiating the control from contaminated peanuts (Figure 3). SIMCA was selected for the rapid detection and classification of peanuts contaminated with aflatoxins due to its advantage in minimizing the overfitting problem compared with other common supervised techniques (i.e., support vector machine (SVM), partial least-squares discriminant analysis (PLS-DA), and artificial neural network (ANN)) [48,49]. The optimum numbers of principal components (PCs/factor) for each class under SIMCA model were defined based on explained variance to avoid overfitting and underfitting [48,49]. To generate the FT-IR SIMCA training model for aflatoxins, the spectral range (1799–1446 cm^−1^, 190 data points) that contained more signatures related to aflatoxins was selected. Eight principal components were deployed for the class of peanuts with aflatoxins, and six principal components were utilized for the class of peanuts without aflatoxins in the SIMCA model generated using the FT-IR spectra, explaining 99.4% and 98.8% of the variance, respectively. Peanuts with aflatoxins were well separated from control peanuts, with an interclass distance (ICD) of 3.63 (ICD > 3), suggesting large Euclidian distances between the centers of clusters (important for identification) and yielding distinctive clustering patterns. The SIMCA Coomans plot (Figure 4a) demonstrated the performance of the supervised classification model via determining class membership based on distance from the boundaries (vertical and horizontal lines, 95% confidence limits) of the classes (control peanuts and peanuts spiked with aflatoxins) in the pairwise plot. Control peanuts were well separated from peanuts with aflatoxins based on the unique infrared spectral patterns, and they were all plotted onto the lower right quadrant of the diagram (Figure 4a). Some peanuts with aflatoxins were plotted in the lower left quadrant, representing similarity with control peanuts. However, through the cross-validation analysis, all the aflatoxin-contaminated peanuts were accurately predicted into their assigned class, with no misclassification. The discriminating power plot (Figure 4b) showed the variables having a prevalent impact on sample classification by maximizing the difference between different clusters and minimizing the difference between samples within clusters [50]. The results showed that bands centered at 1670 cm^−1^ and 1645 cm^−1^ contributed most to detecting peanuts with aflatoxins, which could be attributed to the C=C stretching vibration of the ring structures of aflatoxins [51]. Shen et al. compared spectral profile differences by changing the aflatoxin content in brown rice, and they reported that the pronounced differences at 1644 cm^−1^ might be related to fungal infection in the brown rice, in agreement with our finding [9].

**Figure 5:**
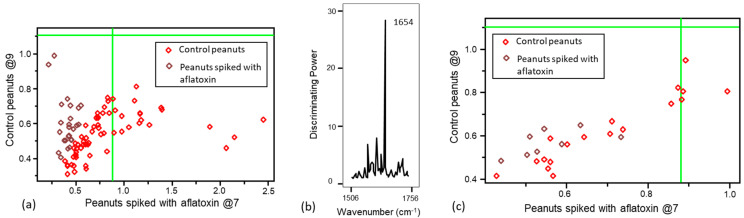
(**a**) SIMCA Coomans plot and (**b**) SIMCA discriminating plot generated from the spectral data collected using a handheld Raman spectrometer (1064 nm), and (**c**) SIMCA Coomans plot for its corresponding external validation set.

To generate the Raman SIMCA training model for aflatoxins, the spectral range (1506–1756 cm^−1^, 56 data points) that contained more signatures related to aflatoxins was selected. The SIMCA classification analysis using Raman spectra showed limited ability for discriminating peanuts spiked with aflatoxins below 100 ppb; therefore, the models were only generated by including spiked peanuts with aflatoxin levels ≥ 100 ppb (100, 200 and 400 ppb). The classification model employed nine principal components to explain 89.5% of the variance in the class of peanuts spiked with aflatoxins, and seven principal components were utilized to explain 93.0% of the variance in the class of peanuts without aflatoxin. The Coomans plot showed a limited separation of the two classes (in the lower left quadrant of Figure 5a), and control peanuts and peanuts spiked with aflatoxins only had an ICD of 0.6 (ICD < 3), with no misclassification, showing that limited Raman spectral features were resolved for identifying aflatoxins. The discriminating power plot (Figure 5b) showed that bands centered at 1654 cm^−1^, associated with C=O stretching vibrations in the aflatoxins’ structure, were important for differentiating and detecting peanuts with aflatoxins [52].

### 3.2. Validation of the Pattern Recognition Models

After verifying that the supervised SIMCA training models correctly classified peanuts contaminated with aflatoxins, we challenged the models, to evaluate their performance, by introducing an independent external validation set (Figure 4c and Figure 5c). The predictive capability of these models was evaluated using sensitivity, specificity, and accuracy (correct classification rate). Sensitivity was used to evaluate the capability of the classification model to identify single peanut kernels with aflatoxins. Specificity was used to evaluate the ability of the model to detect the peanuts without aflatoxins, while accuracy was used to determine the ability of the model to predict peanuts (with and without aflatoxins) into their actual class [53]. All 20 validation peanut kernels were accurately predicted to the corresponding class (n_true positive_ = 10, n_false negative_ = 0, n_false positve_ = 0, n_true negative_ = 10), resulting in 100% accuracy, 100% sensitivity, and 100% specificity (Table 2).

The performance of the supervised SIMCA model generated with Raman data was assessed using an external validation set with 8 peanuts with aflatoxins (100, 200, and 400 ppb) and 18 peanuts without aflatoxins, illustrating 80.8% accuracy, 62.5% sensitivity, and 88.9% specificity (n_true positive_ = 5, n_false negative_ = 3, n_false positve_ = 2, n_true negative_ = 16) in their predictions. This revealed that the Raman model had an adequate ability to differentiate peanuts without aflatoxins but had a limited capability to identify the peanuts with aflatoxins. The fingerprinting information of aflatoxins might not be easily extracted and identified from the Raman spectrum because more interference from other chemical functional groups in the cotyledons was present in the Raman spectrum than in the FT-IR spectrum (deeper penetration of Raman). This difference could limit the ability to detect aflatoxins on kernel surfaces using Raman spectra.

An overview of previous studies performed using FT-IR and Raman spectroscopy is summarized in Table 3, comparing the performance of our models with that of the models reported in the literature. Previous FT-IR studies for classifying aflatoxin-contaminated peanuts were conducted using benchtop FT-IR instrument equipped with ATR accessory or with a diffuse reflectance sample holder. Overall, the models that we obtained in this study using a portable FT-IR instrument provided similar or even superior performance in terms of accuracy, sensitivity, and specificity. Comparing our results to those reported by Lee et al. (who used homogenized ground maize samples and a benchtop Raman instrument equipped with 785 nm laser, and analyzed the data with different pattern recognition techniques) showed that our model yielded higher accuracy (correct classification rates) than their model generated by PLS-DA but slightly lower accuracy than the ones generated using KNN and LDA [53,54]. Moreover, the low aflatoxin concentration range (30–400 ppb) utilized for this study would cover almost all possible early-stage-contaminated peanuts in the peanut production industry and commercial market, providing a rapid and nondestructive pass/fail screening approach. In addition, to the best of our knowledge, our study is the first to report a nondestructive classification approach to detect single aflatoxin-contaminated peanut kernels using field-portable vibrational spectroscopy instruments.

## 4. Conclusions

Our study demonstrated that both field-deployable FT-IR and Raman instruments have the potential to nondestructively and rapidly classify/identify aflatoxin-contaminated peanuts. The developed chemometric (SIMCA) models based on FT-IR and Raman spectral data demonstrated robust and excellent predictive ability, as supported by the results of internal and external validations, for identifying the peanuts contaminated with aflatoxin. In our study, the performance of the FT-IR spectroscopic method was superior that of the Raman method in terms of detection limit (30 ppb for FT-IR and 100 ppb for Raman) and model prediction. Our FT-IR prediction model provided similar or even superior performance to that reported using benchtop infrared systems. Our Raman model showed comparable performance in terms of accuracy to previous methods using benchtop Raman equipped with a 785 nm laser. With the development of this classification approach using *Aspergillus*-infected peanuts in a future study, these state-of-art field-deployable instruments could be used for the real-time monitoring and rapid identification of early-stage-contaminated samples in terms of penetration depth, which would greatly benefit consumers and the food industry, ensuring food safety and quality.

## Figures and Tables

**Table 1:** The aflatoxin content verification analysis results of randomly selected peanuts after their spectral acquisition.

Spiked Concentration (ppb)	Sample Number	LC-MS/MS Results (ppb)				
		Min	Max	Mean	STD	SE
50	4	36.49	61.99	46.28	11.19	5.60
100	4	123.02	141.79	130.57	8.15	4.08
200	4	194.11	248.84	227.18	23.52	11.76
400	4	341.35	394.05	372.34	24.33	12.17

**Table 2:** Statistical performance results of SIMCA models obtained from portable FT-IR and handheld Raman (1064 nm) spectral data.

Model Types	Accuracy (%)	Sensitivity (%)	Specificity (%)
FT-IR	100	100	100
Raman	80.8	62.5	88.9

**Table 3:** Overview of previous studies performed using FT-IR and Raman spectroscopy to detect aflatoxin content with bulk crop samples.

Sample	Aflatoxins	Instrument	Chemometrics	Results	Reference
Ground peanut paste	Aflatoxin B1 (<10,624 ppb)	Benchtop FT-IR with ATR accessory	Bagged decision tree	Accuracy = 77%	[55]
Brown rice	Aflatoxins (B1, B2, G1, G2) (<2406 ppb)	Benchtop FT-IR with ATR accessory	LDA ^a^	Accuracy = 90.6%	[9]
Ground maize	Aflatoxins (B1, B2, G1, G2) (<1206 ppb)	Benchtop FT-IR with a diffuse reflectance holder	KNN ^b^ LDA PLS-DA ^c^	KNN: CCR ^d^ = 84% LDA: CCR ^d^ = 84% PLS-DA: CCR = 72%	[53]
Ground maize	Aflatoxins (B1, B2, G1, G2) (<1206 ppb)	Benchtop Raman (785 nm)	LDA PCDA ^e^ PLS-DA	LDA: False negative rate = 0% PCDA: False negative rate = 13.3% PLS-DA: False negative rate = 6.7%	[54]
Ground maize	Aflatoxins (B1, B2, G1, G2) (<1206 ppb)	Benchtop Raman (785 nm)	KNN LDA PLS-DA	KNN: CCR = 91.4% LDA: CCR = 91.4% PLS-DA: CCR = 53.4%	[53]

^a^ LDA: linear discriminant analysis^; b^ KNN: k-nearest neighbors; ^c^ PLS-DA: partial least-squares discriminant analysis; ^d^ CCR: correct classification rate; ^e^ PCDA: principle component discriminant analysis.

## Data Availability

Data is contained within the article.
